# Assessment of basic reproductive number for COVID-19 at global level: A meta-analysis

**DOI:** 10.1097/MD.0000000000025837

**Published:** 2021-05-07

**Authors:** Cheng-Jun Yu, Zi-Xiao Wang, Yue Xu, Ming-Xia Hu, Kai Chen, Gang Qin

**Affiliations:** a Department of Internal Medicine, Medical School, Nantong University, Nantong, China; b Department of Computer Science, New York Institute of Technology, New York, NY, USA; c School of Pharmacy, Macau University of Science and Technology, Macau; d Department of Epidemiology and Biostatistics, School of Public Health, Nantong University, Nantong, China.

**Keywords:** 2019 novel coronavirus disease (COVID-19), basic reproduction number (R_0_), meta-analysis

## Abstract

**Background:**

There are large knowledge gaps regarding how transmission of 2019 novel coronavirus disease (COVID-19) occurred in different settings across the world. This study aims to summarize basic reproduction number (R_0_) data and provide clues for designing prevention and control measures.

**Methods:**

Several databases and preprint platforms were retrieved for literature reporting R_0_ values of COVID-19. The analysis was stratified by the prespecified modeling method to make the R_0_ values comparable, and by country/region to explore whether R_0_ estimates differed across the world. The average R_0_ values were pooled using a random-effects model.

**Results:**

We identified 185 unique articles, yielding 43 articles for analysis. The selected studies covered 5 countries from Asia, 5 countries from Europe, 12 countries from Africa, and 1 from North America, South America, and Australia each. Exponential growth rate model was most favored by researchers. The pooled global R_0_ was 4.08 (95% CI, 3.09–5.39). The R_0_ estimates for new and shifting epicenters were comparable or even higher than that for the original epicenter Wuhan, China.

**Conclusions:**

The high R_0_ values suggest that an extraordinary combination of control measures is needed for halting COVID-19.

## 1 Introduction

In January 2020, the general public became aware of an outbreak of a novel coronavirus strain, now termed severe acute respiratory syndrome coronavirus 2 (SARS-CoV-2) which had been affecting Wuhan city, China. The disease has been spreading rapidly worldwide, leading the World Health Organization (WHO) to declare a pandemic on March 11, 2020. While majority of cases have been relatively mild outside of Wuhan,^[1]^ a lot of uncertainties remain about the severity of the 2019 novel coronavirus disease (COVID-19) on a per-case basis. Furthermore, higher-than-normal spread rates expected from this virus may result in population-level severe morbidity and mortality even if the case-fatality ratio remains low.

There exist large knowledge gaps regarding how transmission of SARS-CoV-2 occurred in different settings across the world. The basic reproduction number (R_0_) is one of the fundamental and most often used metrics that describes the contagiousness or transmissibility of the infectious agent at the beginning of an epidemic. The proportion of the population needed to be vaccinated for the elimination of an infection can be based on R_0_ values.^[2]^ Estimating R_0_ is a requisite for designing prevention and control measures for infectious diseases such as COVID-19.

Although many researchers estimated the reproductive number of COVID-19, their results, as well as stages of infection, measurement methods, and applied preventive interventions differ substantially across the studies. Considering the variability of the reproductive numbers among the countries, we attempt to summarize available R_0_ estimates of COVID-19 at the global level. The pooled statistical findings might help to characterize the spread of the disease and inform public health policy.

## 2 Methods

### 2.1 Search strategy

Our study conforms to the Preferred Reporting Items for Systematic Reviews and Meta-analysis guidelines.^[3]^ We retrieved literature from the PubMed, EmBase, China National Knowledge Infrastructure (Chinese), WanFang (Chinese) database, and BioRxiv, MedRxiv, arXiv preprint platforms in August, 2020. The search terms included: (“novel coronavirus” or “SARS-CoV-2” or “2019 novel coronavirus disease” or “COVID-19”) and (“basic reproduction number” or “R_0_” or “transmission” or “epidemic dynamics”). To identify additional studies, we reviewed and hand searched the references of important articles.

### 2.2 Study selection

Two independent authors screened the titles and abstracts for relevance. Articles were evaluated for inclusion according to the following criteria: described the early epidemic dynamics of COVID-19 in the country or region; reported basic reproduction number based on daily data of COVID-19 case counts; and presented in English or Chinese language.

Nonhuman and laboratory studies were excluded, as well as studies merely reporting time-dependent reproductive number. No exclusions were made for modeling methods used for R_0_ estimation.

### 2.3 Data extraction

The name of the first author, country or region, estimation period, measurement method, the estimated R_0_ value (with certain confidence interval [CI]) and digital object identifier were extracted from the articles. All studies that estimated R_0_ for COVID-19 were used for systemic review, while only those with 95% CI were entered into the meta-analysis.

### 2.4 Statistical analysis

We stratified the analysis by prespecified modeling method to make the R_0_ values comparable, and by country/region to examine if R_0_ estimates differed across the world. The average R_0_ values were pooled using a random-effects model. The *I*^2^ values were used for heterogeneity evaluation. A threshold of *I*^2^ ≥ 50% indicated high heterogeneity. Publication bias was assessed with Begg and Egger tests, as well as the funnel plots visually. Moreover, sensitivity analysis was performed using a 1-study-removed analysis.

All statistical analyses were conducted using Stata software (version 15.0, StataCorp, TX, USA). Statistical significance was defined as a *P* value < .05. The map was performed using ArcGIS software (version 10.6, Esri, USA).

## 3 Result

### 3.1 Study selection and characteristics

**Figure 1:**
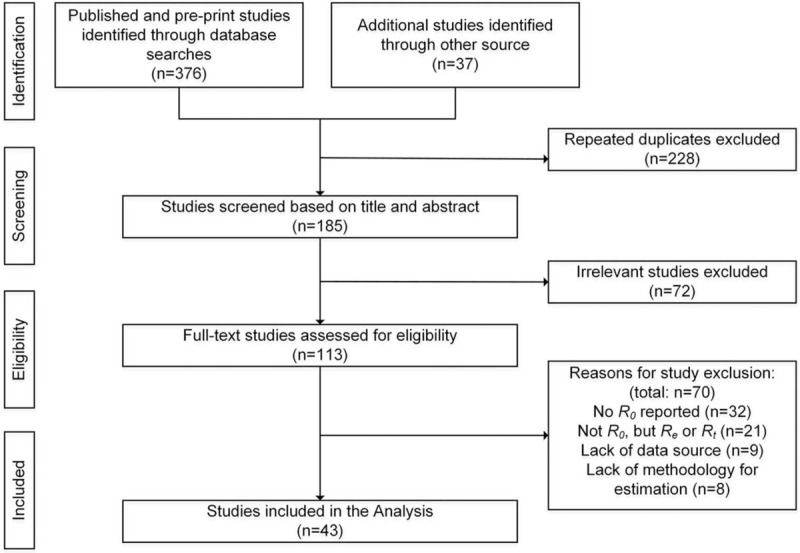
Flow diagram for study selection.

Our database search led to 413 studies, 228 of which were duplicates, and 72 of which were deemed irrelevant based on review of their titles and abstracts. Of the remaining 113 studies, 32 did not report the reproduction number, 21 reported effective reproduction number (Re) or real-time reproduction number (Rt) rather than R_0_, 9 lacked data source and 8 lacked methodology for estimation. Finally, a total of 43 studies were included for the analysis (Fig. 1).^[4–46]^

From the 43 studies (Tables 1 and 2), 61 estimates of R_0_ values were exacted, majority (39) of which were for China (including for Wuhan city specifically). The remaining estimates were presented for Korea (6), United States (3), Italy (2), Iran (2), followed by Japan (1), India (1), The United Kingdom (1), France (1), Germany (1), Spain (1), Brazil (1), Australia (1), and top 12 countries in Africa (1).

**Table 1:** R_0_ estimates for cities and provinces within China.

Author	Location	Estimation period	Methods	R_0_	95% CI
Li JH^[7]^	Wuhan	January 10–23, 2020	EGR	5.54	5.07–6.06
Liu T^[8]^	Wuhan	By February 7, 2020	EGR	4.40	4.30–4.60
Liu T^[8]^	China	By February 7, 2020	EGR	4.50	4.40–4.60
Sanche S^[9]^	Wuhan	January 15–30, 2020	EGR	5.70	3.80–8.90
Song QQ^[10]^	China	January 15–31, 2020	EGR	3.74	3.63–3.87
Wang Y^[5]^	China	Jan 17 to February 8, 2020	EGR	3.49	3.42–3.58
Zhao QY^[11]^	Wuhan	By January 23, 2020	EGR	5.70	3.40–9.20
Zhao S^[12]^	China	January 10–24, 2020	EGR	2.24	1.96–2.55
Zhao S^[12]^	China	January 10–24, 2020	EGR	3.58	2.89–4.39
Zhao S^[13]^	Wuhan	January 1–15, 2020	EGR	2.56	2.49–2.63
Li JH 7	Wuhan	January 10–23, 2020	SEIR	3.55	2.97–4.21
Read J^[14]^	China	January 1–22, 2020	SEIR	3.11	2.39–4.13
Shen MW^[15]^	China	December 12, 2019 to January 22, 2020	SEIR	4.71	4.50–4.92
Song QQ^[10]^	China	January 15–31, 2020	SEIR	3.91	3.71–4.11
Tang B^[16]^	China	By January 22, 2020	SEIR	6.47	5.71–7.23
Zhou T^[17]^	China	By 25 January, 2020	SEIR	2.80–3.30 (3.05)	/
Zhou WK^[18]^	China	By January 10, 2020	SEIR	5.32	/
Cao ZD^[19]^	China	By January 23, 2020	SEIRDC	4.08	
Chen TM^[20]^	Wuhan	December 7, 2019 to January 1, 2020	SEIAR	3.58	/
LI Y^[21]^	China	By January 23, 2020	SEIQR	5.60	/
Li JH^[7]^	Wuhan	January 10–23, 2020	MLE	2.65	2.64–2.67
Song QQ^[10]^	China	January 15–31, 2020	MLE	3.16	2.90–3.43
Tang B^[22]^	China	January 1–23, 2020	MLE	3.80	3.50–4.20
Tang B^[22]^	Guangdong	January 19–31, 2020	MLE	3.00	2.60–3.30
Wang Y^[5]^	China	January 17 to February 8, 2020	MLE	2.99	2.93–3.06
Jung SM^[23]^	China	By January 24, 2020	Epidemic growth model	2.10	2.00–2.20
Jung SM^[23]^	China	By January 24, 2020	Epidemic growth model	3.20	2.70–3.70
Li JH^[7]^	Wuhan	January 10–23, 2020	Sequential Bayesian method	1.68	1.09–2.33
Wang Y^[5]^	China	January 17 to February 8, 2020	Sequential Bayesian method	2.80	2.42–3.15
Li JH^[7]^	Wuhan	January 10–23, 2020	Time dependent reproduction number	5.95	4.96–7.03
Wang Y^[5]^	China	January 17 to February 8, 2020	Time dependent reproduction number	4.48	4.26–4.71
Cao ZD^[24]^	Wuhan	By January 23, 2020	Geo-stratified debiasing estimation framework	3.24	/
Chinazzi M^[25]^	China	By January 23, 2020	GLEAM and SLIR	2.57	2.37–2.78 (90% CI)
Li Q^[26]^	China	By January 22, 2020	Fitted transmission model with zoonotic infection	2.20	1.40–3.90
Du ZW^[27]^	Wuhan	By January 22, 2020	Hierarchical model	1.90	1.47–2.59
Imai N^[28]^	China	By January 18, 2020	Mathematical model	1.50–3.50	/
Majumder M^[29]^	Wuhan	December 8, 2019 to January 26, 2020	Incidence Decay and Exponential Adjustment (IDEA) model	2.00–3.10 (2.50)	/
Wu J^[30]^	Wuhan	December 31, 2019 to January 28, 2020	Markov Chain Monte Carlo methods	2.68	2.47–2.86
Riou J^[31]^	China	By January 18, 2020	Stochastic simulations of early outbreak trajectories	2.20	1.40–3.80 (90% HDI)

CI = confidence interval, EGR = exponential growth rate, GLEAM = global epidemic and mobility model, HDI = high-density interval, MLE = maximum likelihood estimation, SEIAR = susceptible, exposed symptomatic, infectious asymptomatic, infectious removed, SEIQR = susceptible, exposed, infected but not hospitalized, infectious and isolated recovered, SEIR = susceptible-exposed-infected-removed, SEIRDC = SEIR with death cumulative, SLIR = susceptible latent infectious recovered.

**Table 2:** Country-level R_0_ estimates across the world except China.

Author	Location	Estimation period	Methods	R_0_	95% CI
de Souza W^[32]^	Brazil	February 25 to March 19, 2020	EGR	3.10	2.40–5.50
Dwivedi L^[33]^	India	March 14 to April 3, 2020	EGR	2.56	/
Ki M^[34]^	Korea	January 20 to February 10, 2020	EGR	0.48	0.25–0.84
Musa SS^[4]^	Africa	March 1–19, 2020	EGR	2.37	2.22–2.51
Yuan J^[35]^	France	February 23 to March 9, 2020	EGR	6.32	5.72–6.99
Yuan J^[35]^	Germany	February 21 to March 9, 2020	EGR	6.07	5.51–6.69
Yuan J^[35]^	Italy	February 23 to March 9, 2020	EGR	3.27	3.17–3.38
Yuan J^[35]^	Spain	February 19 to March 9, 2020	EGR	5.08	4.51–5.74
Choi S^[36]^	Korea	January 20 to February 17, 2020	SEIR	0.56	0.51–0.60
Dropkin G^[37]^	United Kingdom	January 30 to March 31, 2020	SEIR	6.94	6.52–7.39
Kuniya T^[38]^	Japan	January 15 to February 29, 2020	SEIR	2.60	2.40–2.80
D’Arienzo M^[39]^	Italy	January 25 to March 12, 2020	SIR	2.43–3.10	/
Khosravi A^[40]^	Iran	February 20 to March 5, 2020	MLE	2.74	2.10–3.40
Tang B^[22]^	Korea	January 23 to March 2, 2020	MLE	2.60	2.50–2.70
Muniz-Rodriguez K^[41]^	Iran	February 19 to March 1, 2020	Generalized growth model	4.40	3.90–4.90
Shim E^[6]^	Korea	January 20 to February 26, 2020	Generalized growth model	1.50	1.40–1.60
Fellows IE^[42]^	United States	January 22 to March 14, 2020	Sequential Bayesian method	2.37	2.22–2.52
Gunzler D^[43]^	USA	By March 17, 2020	Sequential Bayesian method	4.02	3.69–5.15
Zhuang Z^[44]^	Korea	January 31 to March 1, 2020	Stochastic model	2.60	2.30–2.90
Zhuang Z^[44]^	Korea	February 5 to March 1, 2020	Stochastic model	3.20	2.90–3.50
Rockett R^[45]^	Australia	January 21 to March 28, 2020	Agent-based model	2.27	/
Ives AR^[46]^	New York state, USA	February 26 to April 20, 2020	Time-varying autoregressive state-space model	6.40	4.30–9.00

CI = confidence interval, EGR = exponential growth rate, MLE = maximum likelihood estimation, SEIR = susceptible-exposed-infected-removed, SIR = susceptible- infected-removed.

### 3.2 Methodological approaches used by the selected studies

As shown in Table 1, the evaluation techniques or models for R_0_ estimation were diverse. Exponential growth rate (EGR) model, susceptible-exposed-infected-removed (SEIR) model or other susceptible-infected-removed-based models, maximum likelihood estimation (MLE) model were the top 3 methods favored by researchers. Other methods include generalized growth model, sequential Bayesian method, stochastic model, etc. (Tables 1 and 2).

### 3.3 Overview of R_0_ estimates across the world

**Figure 2:**
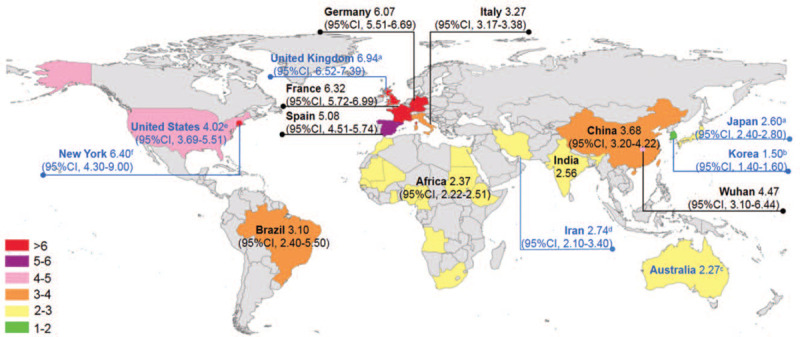
Distribution map of R_0_ estimates (EGR model-based if not specified) ^a^SEIR model; ^b^Generalized growth model; ^c^Agent-based model; ^d^MLE model; ^e^Sequential Bayesian method; ^f^Time-varying autoregressive state-space model. EGR = exponential growth rate, MLE = maximum likelihood estimation, SEIR = susceptible-exposed-infected-removed.

As shown in Figure 2, the R_0_ estimates were conducted for 6 continents, Asia (5 countries), Europe (5 countries), Africa (12 countries), Australia (1 country), North America (1 country), and South America (1 country). The map represented individual R_0_ estimate for 12 countries, and a single R_0_ for Africa covering 12 countries, which was a study at a regional scale.^[4]^ The highest country-level R_0_ estimates are for France (R_0_, 6.32; 95% CI, 5.72–6.99), following Germany (R_0_, 6.07; 95% CI, 5.51–6.69) and Spain (R_0_, 5.08; 95% CI, 4.51–5.74). The R_0_ for Wuhan city, China and New York state, USA were 4.47 (95% CI 3.10–6.44) and 6.40 (95% CI, 4.30–9.00) respectively.

**Figure 3:**
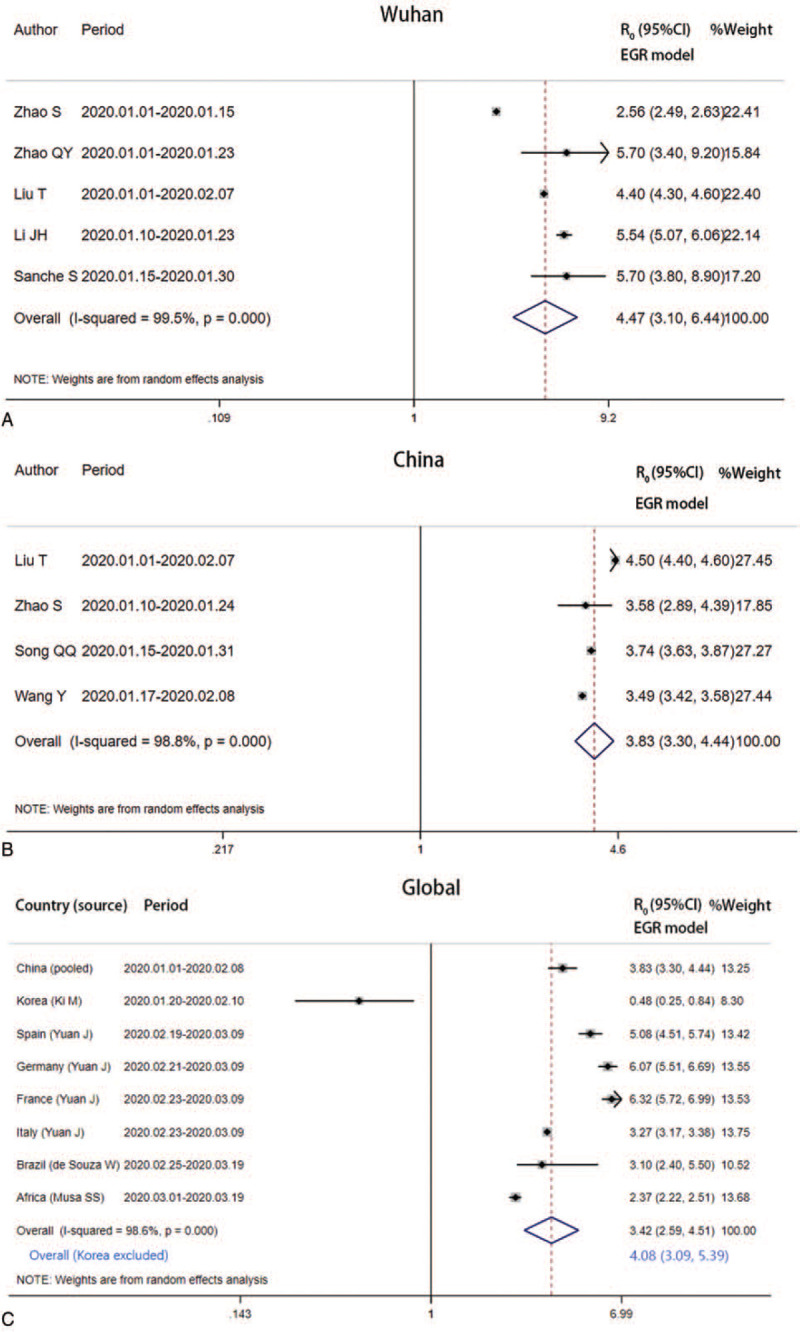
Forest plot of the pooled EGR model-based R_0_ estimates. EGR = exponential growth rate.

After pooling the 5 R_0_ estimates based on EGR models, we got the average R_0_ as 4.47 (95% CI, 3.10–6.44, *I*^2^ = 99.5%) for Wuhan. The pooled R_0_ for China was estimated as 3.83 (95% CI, 3.30–4.44; *I*^2^ = 98.8%). Moreover, combining the pooled result for China and EGR model-based estimates for other 7 countries or regions, we obtained the pooled global R_0_ for COVID-19 was 3.42 (95% CI, 2.59–4.51; *I*^2^ = 98.6%). If the R_0_ estimate for Korea based on data collected before its exponential growth is excluded, the pooled global R_0_ may be 4.08 (95% CI 3.09–5.39) (Fig. 3).

SEIR models have been used for R_0_ estimates for China, Japan, and Korea. The pooled R_0_ for China was estimated as 4.50 (95% CI, 3.71–5.46; *I*^2^ = 96.1%). The pooled Asian R_0_ was 3.50 (95% CI, 1.64–5.36; *I*^2^ = 99.8%) from the meta-analysis (see Figure s1, Supplemental Content, which illustrates the forest plot of the pooled SEIR model-based R0 estimates). Meanwhile, MLE models have also been applied to estimate R_0_ for China, Korea, and Iran. The pooled R_0_ for China was estimated as 3.28 (95% CI, 2.87–3.76, *I*^2^ = 92.3%). The pooled Asian R_0_ estimate was 2.85 (95% CI, 2.41–3.37; *I*^2^ = 81.1%) (see Figure s2, Supplemental Content, which illustrates the forest plot of the pooled MLE model-based R_0_ estimates).

### 3.4 Sensitivity analysis and publication bias

The sensitivity analysis showed that no individual study significantly affected the summarized results of R_0_ (see Figures 3, which illustrates the sensitivity analysis plot of meta-analysis for EGR model-based R_0_ estimates). The publication bias was *P* > .05 in both Begg and Egger tests (data not shown). Figures 4 (Supplemental Content, which illustrates the funnel plot of meta-analysis for EGR model-based R_0_ estimates) showed the Begg funnel plots.

## 4 Discussion

Knowledge of the basic reproductive number is critical for understanding the dynamics of the novel coronavirus disease. As the pandemic progresses in space and time, this needs to be re-evaluated. To the best of our knowledge, this study for the first time summarized the R_0_ values at the global level. This study might help to characterize the spread scale of the disease, and convey a clear message to public health decision makers.

R_0_ is affected by lots of biosocial factors and is estimated by various complex mathematical models. Therefore, the R_0_ values are usually dependent on model structures and assumptions. It is recommended not to compare values based on different models.^[2]^ Based on this review, the EGR model was the most common method addressed by the available studies. It has been more than 3 decades since the development of EGR model which embraces both the infection cycle and the change in number of new case counts within the Lotka–Euler framework.^[47]^ By comparing 4 methods, Wang et al^[5]^ found that EGR model fitted the Chinese COVID-19 data best. Our study calculated pooled R_0_ values with the same method respectively, avoiding the limitation of different modeling. Through this approach we got more comparable estimates than previous meta-analyses.^[48]^

Using the assumption of exponential growth (EGR models), we found R0 values of 4.54 (95% CI 3.18–5.90) and 3.69 (95% CI 3.17–4.21) for Wuhan and China respectively. Compared with severe acute respiratory syndrome (SARS, R_0_ = 3, range 2.2–3.6) and Middle East respiratory syndrome (R_0_ range 0.8–1.3),^[49,50]^ COVID-19 has a higher R_0_, suggesting the novel coronavirus be more contagious and stringent public health strategies be necessary. One modeling study indicated that if R_0_ were above 3.5, even near perfect case isolation and contact tracing would not be sufficient to control COVID-19 outbreaks.^[51]^ Lessons from influenza pandemics also tell us that timely implementation of nonpharmaceutical interventions (NPIs), including infection control, social distancing, small area lockdown, and travel restrictions, is warranted.^[52]^

It is on January 30, 2020 that the WHO declared the COVID-19 a public health emergency of international concern, 1 week after Chinese government launched the unprecedented lockdown in Wuhan epicenter and other 12 cities in Hubei province. Significant decrease in the growth rate of COVID-19 cases within China was reported by us and other researchers.^[53,54]^ In contrast, new epicenters were developing and shifting across the world during the same period. Some epidemiological parameters in countries except China could be different based on control strategies. However, it is clear that the R_0_ values for United States, France, Germany, Italy, and Spain from February to March were comparable or even higher than that of Wuhan. Africa, the last continent to be hit by the COVID-19 pandemic, is expected to be the most vulnerable continent. Interestingly, most of the identified COVID-19 cases in Africa had been imported from Europe and the United States, rather than from the original COVID-19 epicenter China.^[55]^ Maybe, this situation would have been different if the alert call from WHO and the NPI example from China were taken into consideration on time by these authorities.^[56]^

It is worth noting that some estimates of R_0_ were significantly lower for Korea than those for most other countries. The previous 2 studies reporting subexponential growth dynamic in Korea used data collected before February 17, 2020. However, it was on February 19, 2020 when the number of confirmed COVID-19 cases started to increase rapidly. The increased spread of COVID-19 in Korea may be attributed to one superspreading event that had resulted in more than 3900 secondary cases stemming from church services in the city of Daegu.^[6]^

Admittedly, there are several limitations in this study. First, the study covered a small number of world countries and was not enough to cover the geographical dimensions of the continents. Second, the study did not assess meteorological characteristics of a location and demographic characteristics of that location's population which might influence the disease transmission pattern. Last, some articles included for analysis are preprints that might affect the overall quality of the review to some extent.

In conclusion, the relatively high value for R_0_ suggests that an extraordinary combination of control measures is needed for halting COVID-19. Indeed, at the expected transmissibility of the pandemic pathogen, timely NPIs should be implemented before a highly efficacious vaccine could become available. Such efforts will be the key to quell local outbreaks and reduce the risk of further global dissemination.

## Acknowledgments

The authors thank Prof Lei Zhang from Xi’an Jiaotong University Health Science Center for his valuable scientific comments and advices.

## Author contributions

GQ designed the study protocol. CJY, ZXW, and YX did the literature search. The titles, abstracts, and full texts were screened and selected by CJY and MXH. The data were extracted and analysed by CJY, MXH and KC. CJY, ZXW, and YX drafted the manuscript. GQ edited the draft. All authors read and approved the final manuscript.

## Supplementary Material

Supplemental Digital Content

## Supplementary Material

Supplemental Digital Content

## Supplementary Material

Supplemental Digital Content

## Supplementary Material

Supplemental Digital Content

## References

[1] Zhang Y. The epidemiological characteristics of an outbreak of 2019 novel coronavirus diseases (COVID-19) in China. Zhonghua Liu Xing Bing Xue Za Zhi 2020;41:145–51. 32064853 10.3760/cma.j.issn.0254-6450.2020.02.003

[2] Delamater P Street E Leslie T. Complexity of the Basic Reproduction Number (R0). Emerg Infect Dis 2019;25:01–4. 10.3201/eid2501.171901 PMC6302597 30560777

[3] Moher D Shamseer L Clarke M. Preferred reporting items for systematic review and meta-analysis protocols (PRISMA-P) 2015 statement. Syst Rev 2015;4:01. 10.1186/2046-4053-4-1 PMC4320440 25554246

[4] Musa SS Zhao S Wang MH. Estimation of exponential growth rate and basic reproduction number of the coronavirus disease 2019 (COVID-19) in Africa. Infect Dis Poverty 2020;9:96. 32678037 10.1186/s40249-020-00718-y PMC7365306

[5] Wang Y You X Wang Y. Estimating the basic reproduction number of COVID-19 in Wuhan, China. Zhonghua Liu Xing Bing Xue Za Zhi 2020;41:476–9. 32125128 10.3760/cma.j.cn112338-20200210-00086

[6] Shim E Tariq A Choi W. Transmission potential and severity of COVID-19 in South Korea. Int J Infect Dis 2020;93:339–44. 32198088 10.1016/j.ijid.2020.03.031 PMC7118661

[7] Li J Wang Y Gilmour S. Estimation of the epidemic properties of the 2019 novel coronavirus: a mathematical modeling study. medRxiv 2020;preprint. doi: 10.1101/2020.02.18.20024315.

[8] Liu T Hu J Xiao J. Time-varying transmission dynamics of Novel Coronavirus Pneumonia in China. bioRxiv 2020;preprint. doi: 10.1101/2020.01.25.919787.

[9] Sanche S Lin YT Xu C. High contagiousness and rapid spread of severe acute respiratory syndrome Coronavirus 2. Emerg Infect Dis 2020;26:1470–7. 32255761 10.3201/eid2607.200282 PMC7323562

[10] Song Q Zhao H Fang L-Q. Study on assessing early epidemiological parameters of coronavirus disease epidemic in China. Zhonghua Liu Xing Bing Xue Za Zhi 2020;41:461–5. 32113196 10.3760/cma.j.cn112338-20200205-00069

[11] Zhao Q Chen Y Small DS. Analysis of the epidemic growth of the early 2019-nCoV outbreak using internationally confirmed cases. medRxiv 2020;preprint. doi: 10.1101/2020.02.06.20020941.

[12] Zhao S Lin Q Ran J. Preliminary estimation of the basic reproduction number of novel coronavirus (2019-nCoV) in China, from 2019 to 2020: A data-driven analysis in the early phase of the outbreak. Int J Infect Dis 2020;92:214–7. 32007643 10.1016/j.ijid.2020.01.050 PMC7110798

[13] Zhao S Musa SS Lin Q. Estimating the Unreported Number of Novel Coronavirus (2019-nCoV) cases in China in the First Half of January 2020: a data-driven modelling analysis of the early outbreak. J Clin Med 2020;9:388. 10.3390/jcm9020388 PMC7074332 32024089

[14] Read JM Bridgen JR Cummings DA. Novel coronavirus 2019-nCoV: early estimation of epidemiological parameters and epidemic predictions. medRxiv 2020;preprint. doi: 10.1101/2020.01.23.20018549. 10.1098/rstb.2020.0265 PMC8165596 34053269

[15] Shen M Peng Z Xiao Y. Modelling the epidemic trend of the 2019 novel coronavirus outbreak in China. bioRxiv 2020;doi: 10.1016/j.xinn.2020.100048. 10.1016/j.xinn.2020.100048 PMC7831648 33521762

[16] Tang S Wang Z Li Y-S. Estimation of the transmission risk of the 2019-nCoV and its implication for public health interventions. J Clin Med 2020;9:462. 10.3390/jcm9020462 PMC7074281 32046137

[17] Zhou T Liu Q Yang Z. Preliminary prediction of the basic reproduction number of the Wuhan novel coronavirus 2019-nCoV. J Evid Based Med 2020;13:03–7. 10.1111/jebm.12376 PMC7167008 32048815

[18] Zhou W Wang A Xia F. Effects of media reporting on mitigating spread of COVID-19 in the early phase of the outbreak. Math Biosci Eng 2020;17:2693–707. 32233561 10.3934/mbe.2020147

[19] Cao Z Zhang Q Lu X. Estimating the effective reproduction number of the 2019-nCoV in China. medRxiv 2020;preprint. doi: 10.1101/2020.01.27.20018952.

[20] Chen T-M Rui J Wang Q-P. A mathematical model for simulating the phase-based transmissibility of a novel coronavirus. Infect Dis Poverty 2020;9:24. 32111262 10.1186/s40249-020-00640-3 PMC7047374

[21] Li Y Wang LW Peng ZH. Basic reproduction number and predicted trends of coronavirus disease 2019 epidemic in the mainland of China. Infect Dis Poverty 2020;9:article 94. 32678056 10.1186/s40249-020-00704-4 PMC7363992

[22] Tang B Xia F Bragazzi N. Lessons drawn from China and South Korea for managing COVID-19 epidemic: insights from a comparative modeling study. medRxiv 2020;preprint doi: 10.1101/2020.03.09.20033464. 10.1016/j.isatra.2021.12.004 PMC8713134 35164963

[23] Jung S-M Akhmetzhanov AR Hayashi K. Real time estimation of the risk of death from novel coronavirus (2019-nCoV) infection: Inference using exported cases. medRxiv preprint doi:10.3390/jcm9020523. 10.3390/jcm9020523 PMC7074479 32075152

[24] Cao Z Zhang Q Lu X. Incorporating human movement data to improve epidemiological estimates for 2019-nCoV. medRxiv 2020;preprint. doi: 10.1101/2020.02.07.20021071.

[25] Chinazzi M Davis JT Ajelli M. The effect of travel restrictions on the spread of the 2019 novel coronavirus (COVID-19) outbreak. Science 2020;368:395. 32144116 10.1126/science.aba9757 PMC7164386

[26] Li Q Guan X Wu P. Early transmission dynamics in Wuhan, China, of Novel coronavirus-infected pneumonia. N Engl J Med 2020;382:1199–207. 31995857 10.1056/NEJMoa2001316 PMC7121484

[27] Du Z Wang L Cauchemez S. Risk for transportation of 2019 Novel Coronavirus (COVID-19) from Wuhan to Cities in China. medRxiv 2020;preprint. doi: 10.1101/2020.01.28.20019299. 10.3201/eid2605.200146 PMC7181905 32053479

[28] Imai N. <Report 1 Estimating the potential total number of novel Coronavirus cases in Wuhan City, China.>. Imperial College London COVID-19 Response Team 2020.

[29] Majumder M Mandl K. Early transmissibility assessment of a novel coronavirus in Wuhan, China. SSRN Electron J 2020;preprint. doi: 10.2139/ssrn.3524675.

[30] Wu J Leung K Leung G. Nowcasting and forecasting the potential domestic and international spread of the 2019-nCoV outbreak originating in Wuhan, China: a modelling study. Lancet 2020;395:689–97. 32014114 10.1016/S0140-6736(20)30260-9 PMC7159271

[31] Riou J Althaus C. Pattern of early human-to-human transmission of Wuhan 2019-nCoV. bioRxiv 2020;preprint doi: 10.1101/2020.01.23.917351. 10.2807/1560-7917.ES.2020.25.4.2000058 PMC7001239 32019669

[32] de Souza WM Buss LF Candido DDS. Epidemiological and clinical characteristics of the COVID-19 epidemic in Brazil. Nat Hum Behav 2020;4:856–65. 32737472 10.1038/s41562-020-0928-4

[33] Dwivedi L Rai B Shukla A. Assessing the impact of complete lockdown on COVID-19 Infections in India and its burden on public health facilities. Demography India 2020;49:37–50.

[34] Ki M. Epidemiologic characteristics of early cases with 2019 novel coronavirus (2019-nCoV) disease in Republic of Korea. Epidemiol Health 2020;42:e2020007. 32035431 10.4178/epih.e2020007 PMC7285424

[35] Yuan J Li M Lv G. Monitoring transmissibility and mortality of COVID-19 in Europe. Int J Infect Dis 2020;95:311–5. 32234343 10.1016/j.ijid.2020.03.050 PMC7102547

[36] Choi S Ki M. Estimating the reproductive number and the outbreak size of COVID-19 in Korea. Epidemiol Health 2020;42:e2020011–0. 32164053 10.4178/epih.e2020011 PMC7285447

[37] Dropkin G. COVID-19 UK Lockdown Forecasts and R0. 2020. Preprint. doi: 10.3389/fpubh.2020.00256 10.3389/fpubh.2020.00256 PMC7274132 32574315

[38] Kuniya T. Prediction of the epidemic peak of coronavirus disease in Japan, 2020. J Clin Med 2020;9:789. 10.3390/jcm9030789 PMC7141223 32183172

[39] D’Arienzo M Coniglio A. Assessment of the SARS-CoV-2 basic reproduction number, R0, based on the early phase of COVID-19 outbreak in Italy. Biosafety Health 2020;2:57–9. 32835209 10.1016/j.bsheal.2020.03.004 PMC7148916

[40] Khosravi A Chaman R Rohani-Rasaf M. The basic reproduction number and prediction of the epidemic size of the novel coronavirus (COVID-19) in Shahroud, Iran. medRxiv 2020;preprint. doi:10.1017/S0950268820001247. 10.1017/S0950268820001247 PMC7322167 32517845

[41] Muniz-Rodriguez K Fung IC-H Ferdosi SR. Transmission potential of COVID-19 in Iran. medRxiv 2020;preprint doi: 10.1101/2020.03.08.20030643. 10.3201/eid2608.200536 PMC7392448 32320641

[42] Fellows IE Slayton RB Hakim2 AJ. The COVID-19 Pandemic, Community Mobility and the Effectiveness of Non-pharmaceutical interventions: The United States of America, February to May 2020. arXiv 2020;https://arxiv.org/ftp/arxiv/papers/2007/2007.12644.pdf

[43] Gunzler D Sehgal AR. Time-Varying COVID-19 Reproduction Number in the United States. medRxiv 2020;preprint. doi: 10.1101/2020.04.10.20060863.

[44] Zhuang Z Zhao S Lin Q. Preliminary estimating the reproduction number of the coronavirus disease (COVID-19) outbreak in Republic of Korea from 31 January to 1 March 2020. medRxiv 2020;preprint. doi: 10.1101/2020.03.02.20030312. 10.1016/j.ijid.2020.04.044 PMC7194543 32334115

[45] Rockett RJ Arnott A Lam C. Revealing COVID-19 transmission in Australia by SARS-CoV-2 genome sequencing and agent-based modeling. Nat Med 2020;26:1398–404. 32647358 10.1038/s41591-020-1000-7

[46] Ives AR Bozzuto C. State-by-state estimates of R0 at the start of COVID-19 outbreaks in the USA. medRxiv 2020; preprint doi: 10.1101/2020.05.17.20104653

[47] Wallinga J Lipsitch M. How generation intervals shape the relationship between growth rates and reproductive numbers. Proc Biol Sci 2007;274:599–604. 17476782 10.1098/rspb.2006.3754 PMC1766383

[48] He W Yi GY Zhu Y. Estimation of the basic reproduction number, average incubation time, asymptomatic infection rate, and case fatality rate for COVID-19: meta-analysis and sensitivity analysis. J Med Virol 2020;92:2543–50. 32470164 10.1002/jmv.26041 PMC7283745

[49] Cauchemez S Fraser C Van Kerkhove MD. Middle East respiratory syndrome coronavirus: quantification of the extent of the epidemic, surveillance biases, and transmissibility. Lancet Infect Dis 2014;14:50–6. 24239323 10.1016/S1473-3099(13)70304-9 PMC3895322

[50] Lipsitch M Cohen T Cooper B. Transmission dynamics and control of severe acute respiratory syndrome. Science 2003;300:1966–70. 12766207 10.1126/science.1086616 PMC2760158

[51] Hellewell J Abbott S Gimma A. Feasibility of controlling COVID-19 outbreaks by isolation of cases and contacts. Lancet Glob Health 2020;8:e488–96. 32119825 10.1016/S2214-109X(20)30074-7 PMC7097845

[52] Halloran ME Ferguson NM Eubank S. Modeling targeted layered containment of an influenza pandemic in the United States. Proc Natl Acad Sci U S A 2008;105:4639–44. 18332436 10.1073/pnas.0706849105 PMC2290797

[53] Ji T Chen HL Xu J. Lockdown contained the spread of 2019 novel coronavirus disease in Huangshi City, China: early epidemiological findings. Clin Infect Dis 2020;71:1454–60. 32255183 10.1093/cid/ciaa390 PMC7184509

[54] Lau H Khosrawipour V Kocbach P. The positive impact of lockdown in Wuhan on containing the COVID-19 outbreak in China. J Travel Med 2020;27:taaa037. 32181488 10.1093/jtm/taaa037 PMC7184469

[55] Ruth M. Africa braces for coronavirus, but slowly. The New York Times 20202020.

[56] Institute of Labor Economics (IZA), Amuedo-Dorantes C Kaushal N Muchow AN. Is the Cure Worse than the Disease? County-Level Evidence from the COVID-19 Pandemic in the United States. 2020;http://ftp.iza.org/dp13695.pdf

