# Supplementary material for: Assessment of basic reproductive number for COVID-19 at global level: A meta-analysis
Source: Medicine (Baltimore). 2021 May 7;100(18):e25837. doi: 10.1097/MD.0000000000025837 (PMC8104145; doi:10.1097/MD.0000000000025837)
Supplement: Supplemental Digital Content [file medi-100-e25837-s002.doc]

**Figure s2**. Forest plot of the pooled MLE model-based R_0_ estimates

MLE, maximum likelihood estimation

**
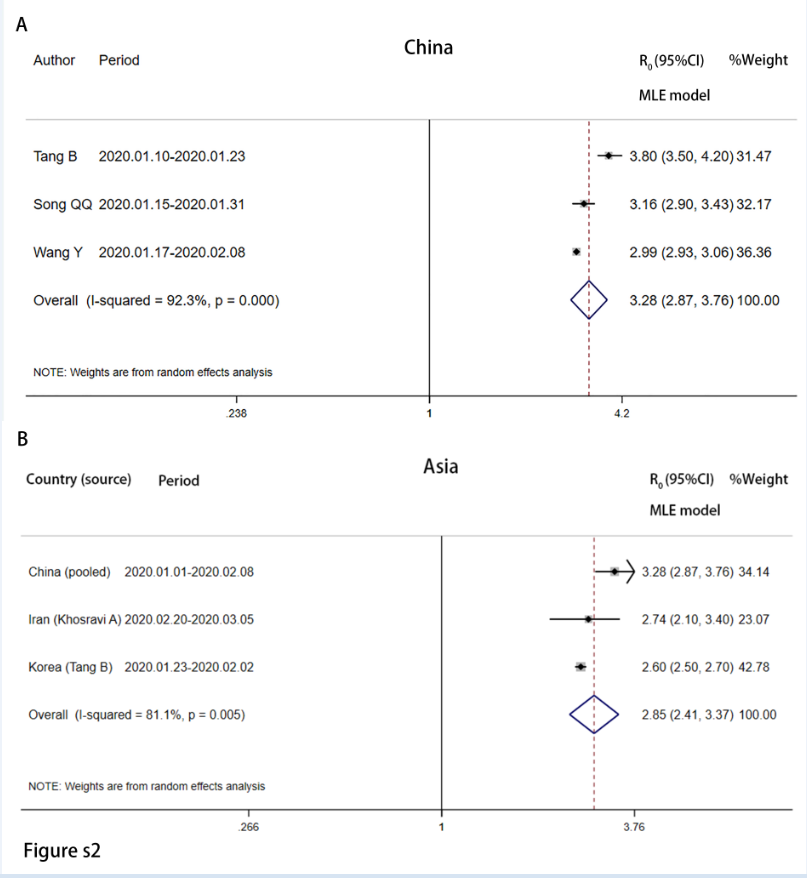
**
