# Supplementary material for: Assessment of basic reproductive number for COVID-19 at global level: A meta-analysis
Source: Medicine (Baltimore). 2021 May 7;100(18):e25837. doi: 10.1097/MD.0000000000025837 (PMC8104145; doi:10.1097/MD.0000000000025837)
Supplement: Supplemental Digital Content [file medi-100-e25837-s003.doc]

**Figrue s3**. Sensitivity analysis plot of meta-analysis for EGR model-based R_0_ estimates

EGR, exponential growth rate

**
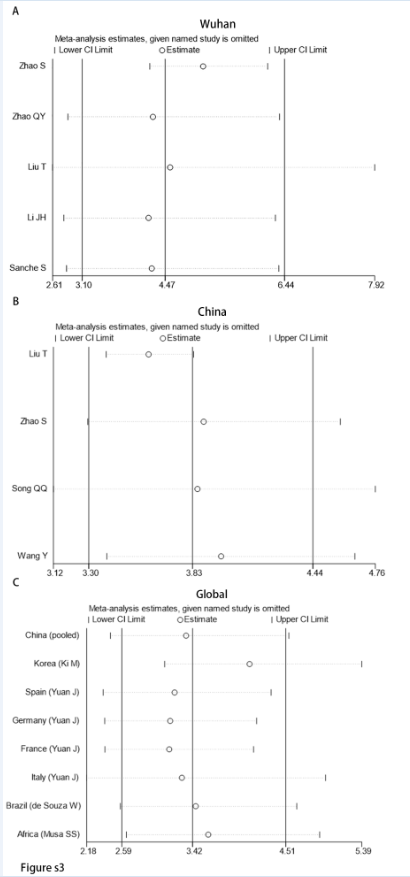
**
